# Announcement: Community Preventive Services Task Force Recommendation for Interventions Engaging Community Health Workers for Diabetes Management

**DOI:** 10.15585/mmwr.mm6636a9

**Published:** 2017-09-15

The Community Preventive Services Task Force (CPSTF) recently posted new information on its website: “Diabetes Management: Interventions Engaging Community Health Workers.” The information is available at https://www.thecommunityguide.org/findings/diabetes-management-interventions-engaging-community-health-workers.

Established in 1996 by the U.S. Department of Health and Human Services, the CPSTF is an independent, nonfederal panel of public health and prevention experts whose members are appointed by the director of CDC. The CPSTF provides information for a wide range of persons who make decisions about programs, services, and other interventions to improve population health. Although CDC provides administrative, scientific, and technical support for the CPSTF, the recommendations developed are those of the CPSTF and do not undergo review or approval by CDC.

